# Core Technological Competence and Competitive Advantage: A Study on Chinese High-Tech SMEs

**DOI:** 10.3389/fpsyg.2022.959448

**Published:** 2022-07-19

**Authors:** Tong Tong, Kashif Iqbal, Azmawani Abd Rahman

**Affiliations:** ^1^Department of Literature, Sichuan Minzu College, Kangding, China; ^2^School of Business and Economics, Universiti Putra Malaysia, Serdang, Malaysia; ^3^School of Business, Shanghai Dianji University, Shanghai, China

**Keywords:** core technical competence, organizational flexibility, competitive advantage, China, SMEs – small and medium sized enterprises

## Abstract

The purpose of this study was to examine the effect of core technology competence on the competitive advantage of high-tech SMEs (small- and mid-sized enterprises in China). Based on the 379 valid responses collected from a survey, structural equation modeling (SEM) was employed to examine the research model. Infrastructure and technology, state-of-the-art technology, innovative research and development (R&D) capability, and organizational flexibility all have a significant impact on the competitive advantage, while infrastructure and technology, state-of-the-art technology, and innovative R&D capability have a significant effect on organizational flexibility. Organizational flexibility plays a mediating role between innovative R&D capability’s effect and competitive advantage. Under the continuous influence of COVID-19, we should promote development from the perspective of strengthening enterprise infrastructure and technology and improving organizational flexibility to gain a competitive advantage. This study reveals the internal relationship between core technology competence, organizational flexibility, and competitive advantage. The results of this study will help us to fully understand the survival status and competitive advantage of high-tech SMEs under COVID-19.

## Introduction

In the modern era of rapid development, information technology has emerged as an essential tool in organizations worldwide. Today, the exponential progress in technological development has enhanced the digital landscape of the leading firms (e.g., high-tech SMEs), allowing organizations to discover newer ways of differentiation (Gomber et al., 2018). High-tech SMEs refer to the many scientific and technological companies that are engaged in scientific and technological research, developing activities, obtaining independent intellectual property rights, and creating high-tech products or services to achieve sustainable development. By the end of 2019, the number of SMEs in China had exceeded 30 million, contributing to more than 50% of the nation’s tax revenue, more than 60% of GDP, more than 70% of technological innovation achievements, and more than 80% of labor employment.

However, the positive development momentum of high-tech SMEs was broken by the sudden outbreak of COVID-19. From February to August 2020, the scale of revocation of high-tech enterprises in China reached 66,000 and the cancelation rate was 4.5% according to the global patent database of the State Intellectual Property Office, which was higher than the overall level of manufacturing and producer services, such that the number and proportion of revocation in high-tech SMEs were significantly higher than those of high-tech large enterprises.

In today’s fast-growing environment, the world’s economies have strived to create significant competitive positioning (Liu and Atuahene-Gima, 2018; Shah et al., 2019a). However, in recent years, the competitive advantage has dynamically evolved with the changing business environment. The evolving realities have allowed the emerging technologies to deliver innovative capabilities, thus establishing a profound foundation for firms’ core competencies and competitive advantage (Nayak et al., 2021). Prior studies have examined enterprise growth, development, and competitive advantage, and core competence has become an important research topic. Barney believed that only having valuable, heterogeneous, non-imitative, and irreplaceable resources can bring lasting competitive advantage to enterprises. Notably, science and technology is the primarily productive force (Scarbrough, 2003). In this regard, core technology competence could be achieved through increasing research and development (R&D) efforts, innovation, and transformation of technological achievements to gain competitive advantages and to show the primacy of technological core competence in high-tech enterprises (Ding and Liu, 2018; Sarfraz et al., 2020).

Undoubtedly, extended digitalization has increasingly forced firms to operate in fierce competition. The organizations functioning in such an environment have fundamentally attained a distinctive edge by embracing advanced technological competence. Core technological competence is the foundation and critical part of scientific and technical enterprises. However, in today’s social environment, the technological upgrading faced by high-tech SMEs has been very rapid. As such, high-tech SMEs must maintain one or several differentiated core technologies (Shah et al., 2019b; Feng et al., 2020). A company with core technology will be more competitive (Voinescu and Moisoiu, 2015). Independent innovation effectively builds the core competitiveness of high-tech SMEs. Therefore, high-tech SMEs should adapt to the external environment and constantly promote the upgrading of products and R&D.

A company’s resources can create excellent performance and transform it into a competitive advantage (Barney, 1991; Tukamuhabwa et al., 2021). Organizational capacity is also a scarce resource for the development of enterprises (Zavolokina et al., 2016). At the same time, organizations should be able to understand their environment and future change trends, increase innovation and R&D of new technologies, occupy the market, and form a productive circular relationship.

China’s technology R&D and transformation of scientific and technological achievements are guaranteed in a good organizational framework. High-tech SMEs are a group of typical technology-driven enterprises; to create more resource advantages than competitors, there must be a structure with a strategic vision to lead the enterprise to success. A flexible organization, through the accumulation of ability and knowledge, can respond to environmental changes at any time and find the most appropriate strategy to obtain a competitive advantage (Shan et al., 2019). Therefore, the current study will focus on the core technical competencies required to gain high-tech SMEs’ competitive advantage through organizational flexibility.

A firm’s technological capabilities strengthen its competence, thus providing a competitive advantage (Shan et al., 2019). Therefore, the purpose of this research was to examine the effect of core technology competence on competitive advantage. We investigated core technology competence in three dimensions based on the actual situation in China. Organizational flexibility is also divided into three dimensions, which are mediating variables. This extends previous research, which independently examined the impact of core technology and organizational flexibility on competitive advantage but found that core technology will bring core rigidity, which will hinder competitive advantage. The results will help us to fully understand how to manage enterprises to survive in the COVID-19 pandemic situation, and how organizational flexibility and technological transformation affect competitive advantage in the current context. Finally, we found that increasing R&D rather than conservative investment during the pandemic is an effective institutional mechanism to increase competitive advantage. This extends existing research that reports on the impact of technological R&D on competitive advantage.

The rest of this review is organized as follows: We propose the research model and hypotheses in section 2. Section 3 reports methodology and data collection. Section 4 reports the finding, and section 5 discusses the findings. We present the study contribution and limitations in section 6.

## Literature Review

### Competitive Advantage

Without a sustainable competitive advantage, enterprises cannot survive continuously. Enterprises with core competitive advantages will be evergreen. Therefore, the research on enterprise competitive advantage has always been the focus of academic interest. However, it has only been in the last decade that China has proposed to revitalize the country through science and technology and has attached importance to the power of science and technology to enhance its comprehensive national strength, with China’s advanced science and technology accounting for only 1.46% of the world. Therefore, there is still a long way to go in exploring the use of technological core competence to enhance the competitive advantage of enterprises in China, especially small- and medium-sized enterprises in science and technology. As the previous research highlights, it is of great significance to find a theory suitable for China’s national conditions that promotes the value of high-tech SMEs.

The main theories of competitive advantage covered in the existing literature are resource-based theory, competence-based theory, knowledge-based theory, and dynamic capability theory.

#### Resource-Based Theory

The resource school believes that each organization is a combination of unique resources and capabilities, which forms the basis of enterprise competitive strategy (Priem and Butler, 2001). According to the resource-based theory, the core technological competence of enterprises is the source for scientific and technological enterprises to obtain a competitive advantage. Enterprise technology core competence is the ability of enterprises to coordinate various resources to perform certain activities (Grant, 1991), because the technology core competence itself has unique characteristics (Barney, 1991), including rareness, non-imitation, and non-sustainability, which is the result of resource deployment and arrangement procedures, a definition that this study adopts. This includes the extent to which the core competence of a specific technology is not held by competitors; the extent to which the core competence of a specific technology cannot be imitated by competitors; and the extent to which the core competence of a specific technology cannot be replaced by other resources or capabilities.

#### Competence-Based Theory

According to the theory of core competence, the core competence of an enterprise is the combination of various skills and the key to obtaining a competitive advantage in the long-term development of the enterprise. However, the limitation of this view is that the organizational process or convention is usually embedded in the enterprise over time (Zollo and Winter, 2002). It rearranges the enterprise’s resources by discarding time-delay resources or reorganizing old resources (Ireland et al., 2003). This means that the core competence of an enterprise is path-dependent in nature (Dierickx and Cool, 1989), because it will affect the decisions made by the enterprise from beginning to end (Zollo and Winter, 2002). This shows that organizational flexibility plays an important role in the construction and development of counter-core competencies. The literature shows that enterprises should obtain a sustainable competitive advantage by combining, absorbing, and transforming basic resources (Cepeda and Vera, 2007; Moliterno and Wiersema, 2007). It is also necessary to build a new operating capacity to reconfigure these new resources (Grant, 1996). New resources often need to be combined, digested, and absorbed before they can become new basic resources of enterprises. However, it is very difficult for enterprises to combine, digest, and absorb resources in the external environment (Zander and Zander, 2005). Galunic and Rodan (1998) contend that the recombination of external resources is concerned with how the knowledge embedded in ability can be integrated and changed with other knowledge bases to create novel business concepts and abilities. The stronger the ability of enterprise reorganization and transformation, the easier it is for the enterprise to internalize external resources into its internal resource base, to maintain a sustainable competitive advantage. A large number of studies propose that the internalization of external resources can often be regarded as the source of the sustainable competitive advantage of enterprises (Fey and Birkinshaw, 2005; Kraaijenbrink et al., 2010). However, with the change of environment, if there is a time lag in the internalized resource base of an enterprise, then that enterprise will not be able to adapt to the dynamic market competition environment. Therefore, the inherent management mode of enterprises may not meet the requirements of enterprises to constantly update their resource base in a dynamic environment.

### The Determinants of Competitive Advantage

According to the resource-based view (RBV) and competence-based view (CBV), the concepts of resource and competence are put forward as the source of competitive advantage. This study agrees with these views, but we also support the complementary evolution process of theories caused by the time and space background of these studies rather than the formation of mutually exclusive theoretical views. These theories emphasize the importance of an industry analysis and argue that the resources and capabilities of enterprises can only be reflected in the industrial competitive environment. Therefore, the strategic management approach of the endogenous theory of competitive advantage can be summarized as industrial environment analysis, enterprise internal resource analysis, formulating competitive strategy, implementing strategy, accumulating strategic resources and establishing core competence matching the industrial environment, winning competitive advantage, and obtaining performance (Conner, 1991), that is, the theoretical paradigm of resource–strategy–performance. In this study, the author contends that, according to the knowledge-based theory and capability-based theory, for industrial enterprises, especially high-tech enterprises, the core competence, particularly the core technical competence, is the fundamental guarantee for the successful implementation of technology strategy.

Once the competitive advantage of knowledge and technology is formed, there will be path dependence and relative stability. This relative stability can easily result in the formation of a rigid core, which causes the enterprise to lose its original competitive advantage. As there is great uncertainty, variability, and complexity in the changing external competitive environment, enterprises are facing confusion and increasing strategic discontinuity, and various factors in the environment are intertwined. Therefore, it is necessary to continuously acquire, integrate, and update the organizational resources and create capabilities that are difficult for competitors to imitate. To achieve this flexible and rapid response, flexible organization and flexible management are needed to promote the renewal of knowledge and technology with the change in environment, so as to obtain a sustainable competitive advantage. As such, according to the theoretical paradigm of resource strategy performance, this study brings the resource-based theory and core competence theory into a theoretical framework.

In recent years, emerging technologies have captured the firms’ interest in gaining a competitive advantage. In this regard, the business core competencies have been viewed as the fundamental construct for adapting and renewing business competitiveness. Technology competency accelerates the momentum of a firm’s journey toward achieving competitive advantage (Shan et al., 2019). Hence, this study puts forward the influence of the core technological capability of high-tech small- and medium-sized enterprises on the competitive advantage of enterprises under the condition of organizational flexibility as an intermediary, contending as follows:

H1: Core technology competence influences competitive advantage.

Core technology competence is a series of knowledge sets used to distinguish the competitive advantage between a specific enterprise and other enterprises. When an enterprise’s core technical competence is regarded as the unique knowledge to identify and solve problems, the core technical competence can often form the most basic sustainable competitive advantage of a specific enterprise and develop more new products to meet various market needs. The research of Bani-Hani and AlHawary (2009) shows a significant positive correlation between enterprises’ core technology competence and sustainable competitive advantage. Accordingly, the literature indicates that an organization’s core competency accelerates the firm’s innovation and competitiveness. In particular, one study shows that technological knowledge competency fosters a firm’s processes, thereby ensuring competitive advantage (Nayak et al., 2021).

In this study, “core technology competence” is defined as the key technologies and related technologies that exist in the whole process of core product formation and service and can be widely used, as well as their coordination and combination capability. Among them, infrastructure and technology refer to the company’s supporting capability and essential technology capability when facing competitive pressure. Significantly, technology infrastructure capability plays an integral role in maintaining a firm’s competitiveness. It facilitates the organization’s internal capabilities, thereby upgrading the firm’s strategic position. IT infrastructure enhances the firm’s ability to compete with peers. It improves the organization’s skill set and core competencies by building a foundation of competitive advantages and benefits. In explaining this notion, one study states that IT infrastructure has emerged as a vital tool for differentiation (Martin-Rojas et al., 2019).

However, in the rapidly changing business environment, firms have focused on maintaining a competitive edge over their peers. This new technological revolution may see the emergence of technology to advance firms’ innovation and competitiveness. The state-of-the-art technological paradigm reengineers the business processes, thereby enabling firms to improve their competitive position. Based on this statement, one study states that today’s high-tech companies have extensively deployed state-of-the-art technologies to overcome the market competition, inevitably achieving a distinctive competitive advantage (Laudon and Laudon, 2020).

However, the literature shows that a firm’s R&D capabilities also play an integral role in fostering its performance, thus establishing a superior competitive benefit. Innovative R&D capability refers to a company’s capability to develop to maintain its future competitive advantage. The technological R&D capabilities advance the firm’s knowledge and innovation process, thereby attaining a better market position. In explaining this phenomenon, one study states that R&D capabilities enable a firm to gain a distinctive market position, thereby promoting its competitive advantage and sustainability (Hwang et al., 2020). Further, based on the aspects mentioned above, the first hypothesis is expanded as follows:

H1 (1): Infrastructure and technology have a significant positive impact on sustainable competitive advantage.

H1 (2): State-of-the-art technology has a significant positive impact on sustainable competitive advantage.

H1 (3): Innovative R&D capability has a significant positive impact on sustainable competitive advantage.

### Organizational Flexibility

Significantly, successful organizations widely adopt flexible means for ensuring a firm’s competitiveness. Organization flexibility elevates the firm’s ability to respond to market uncertainties. It enhances the organizations’ processes by rapidly controlling and maintaining the organizational environment. Business fluctuations are sometimes very chaotic and challenging. As such, one study reveals that organizational flexibility fosters a firm’s processes by effectively managing the market risks (Dubey et al., 2019). In particular, organizational flexibility anchors the firm’s stability, substantially providing a sustainable competitive advantage. In this regard, the study suggests that organizations should adopt organizational flexibility to combat the emerging business challenges, thus gaining a competitive advantage (Kwak et al., 2018).

Based on contingency theory, scholars have discussed the role of organizational flexibility in the environment organization relationship. On the one hand, when enterprises are in a changing environment and the uncertainty and unpredictability of the environmental change trend of enterprises are increasing, flexibility can play an important role in stabilizing enterprise performance and improving enterprise survival probability (Jansen et al., 2005). On the other hand, organizational flexibility can enable organizations to efficiently identify environmental changes, excavate the change list of opportunities and threats, predict new development trends, and carry out corresponding fast and low-cost organizational change actions.

Significantly, successful organizations widely adopt flexible means for ensuring a firm’s competitiveness. Organization flexibility elevates the firm’s ability to respond to market uncertainties. It enhances the organizations’ processes by rapidly controlling and maintaining the organizational environment. Business fluctuations are sometimes very chaotic and challenging. As such, one study reveals that organizational flexibility fosters a firm’s processes by effectively managing the market risk (Van den Bosch et al., 2005). In addition, for organizations with scientific and technological innovation and continuous learning as the main types, organizational flexibility is regarded as a kind of thinking ability. It emphasizes the thinking of the learning system, which is helpful to create a dynamic balance process organizational learning system. Furthermore, enterprises with high performance must have strong flexibility. Organizational flexibility plays a positive role in improving enterprise performance. As such:

H2: Organizational flexibility influences competitive advantage.

A core competence is an action that plays a crucial role in an organization’s “key process,” and organizational flexibility can be used to adjust the allocation of various resources of enterprises. Core competence is a relatively static capability in the process of enterprise development, while organizational flexibility is a relatively dynamic capability. With the development of the market and the progress of science and technology, it is difficult to identify opportunities that cannot be copied, and there will be scarce resources because of technological progress or the need to find alternative resources through artificial synthesis or other factors.

Correspondingly, organizational flexibility refers to the driving force in the development of enterprises, and it is a type of flexibility and adaptability to encourage enterprises to obtain a sustainable competitive advantage. In the technological era, achieving a competitive advantage has become the prime concern of today’s firms. In this regard, technological flexibility ensures the implementation of IT infrastructure (Halpern et al., 2021) and firms’ competitiveness. As such, prior research reveals that technological flexibilities help companies achieve a competitive edge over their market rivals (Agrawal et al., 2021).

Further, with the increase in globalization, dynamic structural flexibilities seek to provide firms with a distinctive competitive advantage. Significantly, structural flexibility plays an incalculable role in facilitating corporate functions. Organizations enjoy favorable market positions due to changes in their structure (Georgewill, 2021). Therefore, firms should focus on reconfiguring structural flexibilities to increase their responsiveness. Altogether, the diverse nature of today’s modern business world has made structural flexibility essential to enhance an organization’s responsiveness to the changing business environment.

However, in the competitive global environment, companies have strongly adopted core competencies to enhance their market position. In this regard, culture has also become prominent as a factor providing firms with a superior competitive advantage. Cultural flexibility influences the organizational environment by transforming and enhancing the firm’s competitiveness (Oliveira, 2021). It encourages organizational innovation and learning, while structural flexibility strengthens internal communication and exchange, and technical flexibility accelerates resource transformation, optimizes enterprise resource allocation, and jointly improves SMEs’ competitive advantage in science and technology. Based on the above analysis, this study puts forward the following hypotheses:

H2 (1): Technological flexibility has a significant positive impact on the sustainable competitive advantage.

H2 (2): Structural flexibility has a significant positive impact on the sustainable competitive advantage.

H2 (3): Cultural flexibility has a significant positive impact on the sustainable competitive advantage.

For small- and medium-sized scientific and technological enterprises, technological innovation is the basis for their survival. An enterprise needs a matching management model to update its products and technologies. While continuously innovating and upgrading its core competencies, it will bring a series of good chain reactions to the organization: First, it triggered the adjustment and upgrading of the industrial structure of enterprises: (1) the direction of industrial adjustment shifted toward the top of the industrial chain—bringing high added value and high profits and (2) promoting productivity, thereby promoting production efficiency.

Second, it promoted the rational distribution of human resources. Employees must strive to learn professional knowledge while the products or technologies of the enterprise are upgrading in the international market. This process can form a virtuous circle of new technological innovation. Third, it broke the rigid management inherent at the department level and made the strategic adjustment of the enterprise timelier.

Based on the above analysis, this study proposes the first hypothesis:

H3 (1): Infrastructure and technology have a significant positive impact on technological flexibility.

H3 (2): State-of-the-art technology has a significant positive impact on technical flexibility.

H3 (3): Innovative R&D capability has a significant positive impact on technological flexibility.

H3 (4): Infrastructure and technology have a significant positive impact on structural flexibility.

H3 (5): State-of-the-art technology has a significant positive impact on structural flexibility.

H3 (6): Innovative R&D capability has a significant positive impact on structural flexibility.

H3 (7): Infrastructure and technology have a significant positive impact on cultural flexibility.

H3 (8): State-of-the-art technology has a significant positive impact on cultural flexibility.

H3 (9): Innovative R&D capability has a significant positive impact on cultural flexibility.

### Mediating Role of Organizational Flexibility Between Core Technical and Sustainable Competitive Advantage

Core competence will be transformed into core rigidity, that is, if managers respond to the current situation with a fixed pattern, it will reduce the enterprises’ competitive advantage. Therefore, it cannot be assumed that a core technological competence will bring a competitive advantage. A mediator is needed to enhance the relationship between core technology competence and competitive advantage (Volberda, 1996). It is considered that organizational change is synchronized with environmental change, and organizational flexibility includes organizational management ability and response-ability. Therefore, flexibility can play an important role in stabilizing enterprise performance and improving enterprise survival probability. Technology is the potential, the seed, while the organization is the soil for how technology develops and transforms, ultimately determining whether the potential can be transformed into productivity (Zhang et al., 2016). Therefore, organizational flexibility eliminates the negative impact of core rigidity on competitive advantage and bolsters performance advantage through continuous innovation. Infrastructure flexibility enables firms to compete in the competitive environment. It develops a system that makes companies respond to market changes. Meanwhile, IT capabilities support the firm’s structure. Also, the technology infrastructure accelerates the firm’s R&D capabilities, thereby formalizing its development process (Zawislak et al., 2018). Therefore, technological infrastructure is an essential tool that fosters technological flexibility.

Undoubtedly, flexibility is central to achieving successful development. In recent years, structural flexibility has helped companies tap new business opportunities, thus enhancing their operations. A firm’s structural flexibility enables it to expand its operations by strengthening its infrastructure development. In an uncertain environment, a firm’s technological infrastructure allows it to change its structural processes, thus adjusting to the new environment. It makes the firms anticipate the changes needed in the business operations. In explaining this notion, the prior research states that the flexibility system increases a firm’s value by managing unknown business scenarios (Sánchez-Silva, 2019). Moreover, cultural flexibility also plays an integral role in strengthening the firm’s IT capabilities. As such, the literature suggests that IT infrastructure supported by cultural compatibilities fosters firms’ business processes (Shahzad et al., 2020). Based on the above analysis, the following hypotheses are put forward:

H4 (1): Technology flexibility mediates the relationship between infrastructure and technology and competitive advantage.

H4 (2): Technology flexibility mediates the relationship between state-of-the-art technology and competitive advantage.

H4 (3): Technology flexibility mediates the relationship between innovative R&D capability and competitive advantage.

H4 (4): Structural flexibility mediates the relationship between infrastructure and technology and competitive advantage.

H4 (5): Structural flexibility mediates the relationship between state-of-the-art technology and competitive advantage.

H4 (6): Structural flexibility mediates the relationship between innovative R&D capability and competitive advantage.

H4 (7): Cultural flexibility mediates the relationship between infrastructure and technology and competitive advantage.

H4 (8): Cultural flexibility mediates the relationship between state-of-the-art technology and competitive advantage.

H4 (9): Cultural flexibility mediates the relationship between innovative R&D capability and competitive advantage.

**FIGURE 1:**
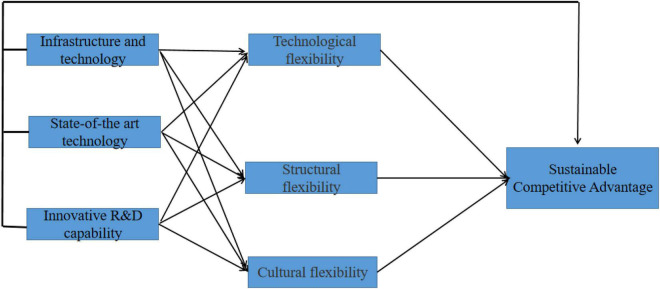
Concept model.

Figure 1 represents the study’s conceptual framework.

## Methodology

This study adopts the empirical research method, and the required data were collected by questionnaire. In the process of questionnaire analysis, we deleted the unreasonable options and improved the questionnaire. A seven-point Likert scale was selected for the questionnaire items measurement ranging from 1 to 7 (1—extremely disagree, 2—disagree, 3—slightly disagree, 4—uncertain, 5—slightly agree, 6—agree, and 7—very agree). The formal questionnaire consists of three parts: the first part is the background of the questionnaire—this part mainly explains the rationality of the survey purpose; the second part is the basic information of the survey enterprise and the personal information of the participants—to help confirm whether the received questionnaire is representative, and the third part is the main part of the questionnaire, with a total of 37 items, including core technology, organizational flexibility environmental uncertainty, enterprise performance, and four categories.

Core technical competence is the independent variable in this study. The measurement items of core technical competence are based on the studies of Long and Vickers-Koch (1995). The core technical competence variable was measured on a scale of 15 items. The core technical competence is divided into three dimensions: infrastructure and technology, state-of-the-art technology, and innovative R&D capability. Organizational capability is a mediating variable in this study, and the scale was adopted from the studies of Jansen et al. (2005) and Zahra et al. (2006). We can observe that high-tech SMEs are keen on technological upgrading, strategic cooperation, and creating competitive advantages with product technology innovation. Therefore, this study considers R&D investment, enterprise personnel size, and enterprise age as control variables. Competitive advantage scale was taken from the study of Chen (2008).

The sample size should be kept at more than 150 in the data analysis with the structural equation model (Anderson and Gerbing, 1988), and the ratio of measurement items to respondents should be in the range of 1:5 to 1:10. Still, a higher ratio is more favorable (Gorsuch, 1997). There are 37 measurement items in this study. According to this standard, the sample size of this study should be at least 210–420 self-built. At the same time, some scholars have proposed that too large a sample size will affect the maximum likelihood estimation. Through the careful consideration of the relevant research, this study determines the sample size as 300–400 to ensure the reliability of the research results.

Here are two ways to distribute the questionnaire: The first way is to distribute and recycle the questionnaire according to the pre-determined research objects, mainly select specific MBA students from universities and qualified middle and senior managers of enterprises selected through personal relations and distribute the questionnaire by directly receiving the enterprise site or e-mail. The second is to entrust the contact person in the organization for questionnaire distribution and collect the questionnaire. In this study, we have adopted an electronic questionnaire, which is more convenient in the current circumstances.

A total of 410 questionnaires were distributed, and 380 questionnaires were received from the participants. We have excluded 66 incomplete questionnaires from the received questionnaires. Finally, 314 valid questionnaires were kept for data analysis. The sample size met the requirements of the data analysis method for large sample data (Christopher Westland, 2010).

## Results

The current study data analysis includes two steps. First, we examined the measurement model to test reliability and validity. Then, we examined the structural model to test the research hypotheses. We examined data normality before data analysis. Table 1 shows the skewness and kurtosis values of each item which are below 2, suggesting good normality (Curran et al., 1996).

**TABLE 1:** Standardized item loadings, AVE, CR, and alpha values.

Factor	Item	Std. Deviation	Skewness	Kurtosis	Standardized loading	AVE	CR	ALPHA
Technological Flexibility (F4)	TF1	1.868	–0.918	–0.169	0.807	0.62	0.82	0.86
	TF2	1.894	–0.871	–0.352	0.882			
	TF3	1.929	–0.913	–0.283	0.768			
	TF4	1.893	–0.905	–0.282	0.654			
	TF5	1.898	–0.971	–0.104	0.835			
Structural Flexibility (F5)	SF1	1.928	–0.954	–0.228	0.599	0.66	0.87	0.82
	SF2	1.914	–0.779	–0.462	0.747			
	SF3	1.899	–0.843	–0.357	0.611			
	SF4	1.883	–0.918	–0.225	0.682			
	SF5	1.752	–0.984	0.117	0.804			
Cultural Flexibility (F6)	CF1	1.770	–0.918	–0.119	0.670	0.83	0.81	0.81
	CF2	1.767	–1.073	0.245	0.751			
	CF3	1.774	–0.929	–0.122	0.816			
	CF4	1.789	–0.862	–0.110	0.693			
	CF5	1.773	–0.993	0.102	0.917			
Innovative R&D Capability (F3)	AI1	1.719	–0.989	0.202	0.942	0.71	0.89	0.68
	AI2	1.755	–1.076	0.273	0.997			
	AI3	1.724	–1.087	0.309	0.954			
	AI4	1.788	–0.999	0.123	0.712			
	AI5	1.712	–1.004	0.072	0.825			
	AI6	1.773	–1.063	0.261	0.839			
Infrastructure And Technology (F1)	BT1	1.755	–1.076	0.273	0.774	0.79	0.80	0.78
	BT2	1.748	–1.028	0.192	0.845			
	BT3	1.767	–1.073	0.245	0.776			
	BT4	1.755	–1.076	0.273	0.825			
	BT5	1.724	–1.087	0.309	0.942			
State-Of-The-Art Technology (F2)	CT1	1.781	–1.050	0.219	0.954	0.83	0.88	0.75
	CT2	1.784	–0.993	0.035	0.885			
	CT3	1.748	–1.028	0.192	0.751			
	CT4	1.709	–1.038	0.167	0.804			
	CT5	1.754	–0.931	0.026	0.670			
Competitive Advantage (F7)	CP1	1.791	–1.074	0.228	0.894	076	0.85	0.80
	CP2	1.767	–1.073	0.245	0.885			
	CP3	1.774	–0.929	–0.122	0.624			
	CP4	1.789	–0.862	–0.110	0.897			
	CI1	1.767	–1.073	0.245	0.870			
	CI2	1.774	–0.929	–0.122	0.710			

First, we checked the reliability through Cronbach’s α coefficient method. The composite reliability (CR) method should be used for measurement testing. In terms of validity tests, this study mainly adopts convergent validity and differential validity (discriminant validity) to measure the validity of the questionnaire. Each AVE (the average variance extracted) is greater than 0.5, reaching the acceptance standard; the CR value meets the threshold requirement of greater than 0.7, indicating that the questionnaire has good convergent validity. The test results of discriminant validity are shown in Table 1. The correlation coefficients of the variables are less than the square root of the AVE value of the variables. To sum up, the scale in this study meets the requirements in terms of reliability and validity.

Second, the main variables included in this study passed the test of reliability and validity. Structural equation modeling was used to analyze the assumed relationship between core technical competence, organizational flexibility, and sustainable competitive advantage. Based on the conceptual model constructed in this study and the corresponding research assumptions, the initial structural equation model’s path diagram is constructed using AMOS22.0. F1 represents infrastructure and technology, F2 represents state-of-the-art technology, F3 represents innovative R&D capability, F4 represents technological flexibility, F5 represents structural flexibility, F6 represents cultural flexibility, and F7 represents sustained competitive advantage. There are seven latent variables in the study. The study’s independent variables are infrastructure and technology, state-of-the-art technology, and innovative R&D capability. The four internal-derived latent variables (dependent variables) of enterprises’ are technological, structural, cultural, and sustainable competitive advantages. The model has residuals of e1–e33 and e34–e37, and their path coefficients are one by default. This study will verify the 15 impact paths set in the initial structural equation model. AMOS22.0 analyzed the initial structural equation model. Table 2 shows the values of average variance extracted and correlation.

**TABLE 2:** Square root of AVE and factor correlation coefficients.

	TF	SF	CF	IT	SCA	IRDC	SCA
TF	1						
SF	0.459**	1					
CF	0.297**	0.421**	1				
IT	0.434**	0.559**	0.633**	1			
IRDC	0.499**	0.542**	0.655**	0.598**	1		
HJ3	0.519**	0.562**	0.659**	0.557**	0.671**	1	
SCA	0.620**	0.663**	0.837**	0.711**	0.791**	0.763**	1

SCA, sustainable competitive advantage; TF, technological flexibility; SF, structural flexibility; CF, cultural flexibility; IT, infrastructure and technology; SCA, state-of-the-art technology; IC, innovative research and development capability. **p < 0.01, ***p < 0.00.

Table 3 presents the results of the relationships between study variables. As shown in Table 3, the path coefficients of the two paths fail to pass the significance test.

**TABLE 3:** Results estimated by AMOS.

			Estimate	S.E.	C.R.	*P*
F4	< ———	F1	0.244	0.094	2.881	0.004
F5	< ———	F1	0.178	0.081	2.243	0.025
F6	< ———	F1	0.255	0.069	3.572	***
F4	< ———	F2	0.143	0.081	2.089	0.037
F5	< ———	F2	0.339	0.073	5.068	***
F6	< ———	F2	0.309	0.062	5.164	***
F4	< ———	F3	0.282	0.096	3.688	***
F5	< ———	F3	0.275	0.083	3.811	***
F6	< ———	F3	0.309	0.071	4.715	***
F7	< ———	F1	0.162	0.038	4.58	***
F7	< ———	F2	0.023	0.034	0.782	0.434
F7	< ———	F3	0.009	0.038	0.283	0.777
F7	< ———	F5	0.228	0.031	7.729	***
F7	< ———	F4	0.296	0.026	11.021	***
F7	< ———	F6	0.56	0.05	12.313	***

**p < 0.01, ***p < 0.00

The empirical results show that the standardized path coefficient of infrastructure and technology and sustainable competitive advantage is 0.243 (*p* < 0.001). The standardized path coefficients of state-of-the-art technology and innovative R&D capability to sustainable competitive advantage are 0.044 (*p* = 0.460) and 0.075 (*p* = 0.277), respectively. This shows that the infrastructure and technology of core technology competence have a significant positive effect on sustainable competitive advantage. In contrast, state-of-the-art technology and innovative R&D capabilities have no direct effect on sustainable competitive advantage.

Infrastructure and technology are the foundation of enterprises. Enterprises with only solid basic skills can survive during the epidemic. Table 4 shows the recommended and actual values of model fit. As shown in Table 4, model fit indices have better actual values than the recommended values.

**TABLE 4:** Model fit values.

Overall fitting coefficient table					
χ^2^/df	RMSEA	GFI	CFI	NFI	IFI
2.4230	0.063	0.814	0.904	0.848	0.905

*χ^2^/df is the ratio between chi-square and degrees of freedom, GFI is the goodness-of-fit index, AGFI is the adjusted goodness-of-fit index, CFI is the comparative fit index, NFI is the normed fit index, NNFI is the non-normed fit index, and RMSEA is root mean square error of approximation.*

It is worth noting that the influence of state-of-the-art technology and innovation and R&D capability on competitive advantage are established under organizational flexibility.

Table 5 shows that organization flexibility fully mediates the effect of state-of-the-art technology, innovation, and R&D capability on competitive advantages. In contrast, organization flexibility partly mediates the effect of infrastructure and technology on competitive advantages.

**TABLE 5:** Bootstrap tests.

Indirect effect path			Non-standardized effect			Ratio (%)	Biased-corrected bootstrap 95%	
			Mediator	Direct Effect	Total Effect		Lower Limit	Upper Limit
BT	SF	YS	0.11**	0.148**	0.636**	17%	0.07	0.14
	CF		0.09**			14%	0.05	0.12
	TF		0.29**			45%	0.23	0.34
CI	SF		0.11**	0.052**	0.572**	19%	0.08	0.14
	CF		0.10**			18%	0.06	0.14
	TF		0.31**			54%	0.26	0.36
AI	SF		0.128**	0.081**	0.649**	20%	0.10	0.16
	CF		0.108**			17%	0.07	0.14
	TF		0.332**			51%	0.29	0.38

**p < 0.01; TF, technological flexibility; SF, structural flexibility; CF, cultural flexibility.

Enterprises’ R&D capability does not directly affect the competitive advantage of enterprises in the study results. This may be due to enterprises’ high expenditure on resources and resources engaged in R&D during the COVID-19. Still, due to the shrinking market demand, this kind of input and output is not proportional to the income, and it is not necessary to carry it out during COVID-19.

Improving enterprise core technology competence can help enhance organizational flexibility, which is an effective way to improve core technology competence. The empirical analysis by constructing a structural equation model shows that the standardized path coefficients between infrastructure and technology and technology flexibility, structural flexibility, and cultural flexibility are 0.334 (*p* < 0.001), 0.290 (*p* < 0.001), and 0.288 (*p* < 0.01), respectively. The standardized path coefficients between state-of-the-art technology and technical flexibility, structural flexibility, and cultural flexibility are 0.185 (*p* = 0.004 < 0.01), 0.276 (*p* < 0.001), and 0.178 (*p* = 0.005 < 0.01), respectively. The standardized path coefficients between innovative R&D capability and technological flexibility, structural flexibility, and cultural flexibility are 0.292 (*P* < 0.001), 0.293 (*P* < 0.001), and 0.264 (*P* < 0.001), respectively. The results show that actively strengthening the core technology construction of SMEs can help cultivate and enhance enterprises’ organizational capacity.

The empirical results show that all dimensions of organizational flexibility positively affect sustainable competitive advantage, which strongly proves the importance of organizational flexibility in enhancing SMEs’ competitive advantage. By constructing a structural equation model for empirical analysis, we draw the following conclusions: The standardized path coefficient between technological flexibility and competitive advantage is 0.279. The standardized path coefficients between structural flexibility and competitive advantage were 0.179 (*P* = 0.008 < 0.01). The standardized path coefficients between cultural flexibility and firm performance are 0.215 and 0.215, respectively (*p* < 0.001). Thus, the dimensions of organizational flexibility have a significant positive impact on sustainable competitive advantage.

## Discussion

Significantly, this empirical study focuses on the degree to which firms’ core competencies provide a competitive advantage. However, to record the study findings, it is vital to understand the results in correspondence with the previous literature. Hence, this section highlights the prime drivers that provide a firm’s competitive advantage. Overall, this section illustrates the study results in light of previous literature reviews.

Undoubtedly, knowledge and technology build an enterprise’s core competencies (Zhou et al., 2018). A firm’s competitive advantage based on technology is the most fundamental tool driving its competitiveness. A firm’s technological innovation strengthens its core competencies, thus fostering competitiveness. In explaining this notion, the research states that the technological management construct adds value to a firm’s processes, thereby achieving a competitive advantage (Dezi et al., 2021). Hence, this prior research supports our study’s findings that reveal a positive relationship between firms’ technological competence and competitive advantage. Hence, this prior research supports our study’s findings that reveal a positive relationship between firms’ technological competence and competitive advantage.

However, in today’s business environment, the technology infrastructure enables firms to outperform their competitors. Therefore, in this regard, the literature states that today’s firms capture the potential markets through their technology and infrastructure competence, thus achieving a competitive advantage (Qosasi et al., 2019). Moreover, in today’s technological era, state-of-the-art technology and R&D capabilities enhance high-tech SMEs’ performance and effectiveness. Perhaps, to attain a competitive advantage, today’s state-of-the-art innovations have helped businesses to improve their operations, thereby gaining an enduring competitive edge (Aceto et al., 2019). Our study results are consistent with the previous literature, meaning H1 (i.e., a, b, and c) is proven.

In particular, the turbulent business environment, unpredictable changes, and technological shifts have forced companies to cope with a dynamic changing environment. In this regard, organizational flexibility has allowed companies to combat the emerging business challenges, thereby profoundly gaining an enduring edge. In explaining this notion, the research states that the direct effect of organizational flexibility potentially provides marginal benefits to companies in the shape of competitive advantage (Koçyiğit and Tabak, 2020). Further, in recent years, technologies have rapidly evolved, thus requiring firms to adapt to the changing market environment (Chester and Allenby, 2019). In correspondence with the prior literature, our results also showed that organizational flexibilities (i.e., technological flexibility, structural flexibility, and cultural flexibility) promote a firm’s enduring competitive advantage. Hence, based on the current findings, we accept the prior assumptions made in H2 (e.g., a, b, and c).

The rise in organizations’ flexibility has inevitably increased scholars’ interest in a competitive advantage. Therefore, understanding this factor requires organizations to focus on different organizational flexibilities (e.g., technological, structural, and cultural) to attain improvised organizational outcomes. As such, the prior research states that a firm’s structural flexibility improves its technology and infrastructure, thus allowing the organization to operate in a turbulent business environment (Liu et al., 2018). Similarly, our study also showed a positive mediating role of organizational flexibilities, leading us to accept H3. Altogether, our study findings have found a significantly positive relationship, thus supporting the prior assumptions made in hypotheses H1, H2, and H3.

This research has a few limitations. First, although the topic selection and related research design are scientific and objective and the expected research objectives and some valuable conclusions are achieved, due to the limitation of the specific background, time, funding, and personal knowledge of COVID-19, the compositional dimensions and measurement of organizational flexibility of high-tech SMEs are still in the exploratory stage. Second, to narrow the scope of the study and make the research more accurate and targeted, this study takes high-tech SMEs as the main research object. To study the accuracy of the results, we used the size of the enterprise and the years of its establishment as a control variable, but there are other control variables that may also have an impact on the results of the study, which could be explored in future research.

## Conclusion

The results of this study demonstrated that, during COVID-19, the only factors that could directly affect the sustained competitive advantage of high-tech SMEs were infrastructure and technology. During COVID-19, SMEs have been eager to seize market share through transformation and innovation; however, many enterprises neither are ready for transformation nor have the ability for cross-domain development, because new products and new technologies are updated too fast, and management cannot keep up with development. Management is busy creating momentum and making money, relaxing the cultivation and transformation of basic business and basic technology, resulting in enterprises not only failing to gain a competitive advantage but also failing to hold their position in the COVID-19 pandemic situation. Overall, with the impact of the global pandemic, the serious recession of the world economy, and the decline in domestic consumption, investment, and export, we should pay more attention to the cultivation of basic technology and skills.

The empirical results show that technological flexibility and structural flexibility have a significant positive impact on sustainable competitive advantage. Therefore, organizational flexibility with high flexibility and agility is an important basis for the formation of competitive advantage and plays a key role in the promotion of sustainable competitive advantage. Under the continuous influence of COVID-19, we should attach great importance to the organization from a strategic perspective. The promotion of flexibility can enhance a high-tech SME’s ability to resist environmental uncertainty and maintain a sustainable competitive advantage. In the COVID-19 pandemic context, high-tech SMEs should form an alliance under the consideration of the strategic goal between the individual enterprise and individuals, exchange complementary resources independently, reach the phased goal of the target product, respectively, obtain the long-term market competitive advantage, and then form a lasting and formal relationship.

## Data Availability Statement

The original contributions presented in this study are included in the article/supplementary material, further inquiries can be directed to the corresponding author.

## Author Contributions

TT wrote the main manuscript. KI proofread and revised the manuscript. AR gave the core idea. All authors contributed to the article and approved the submitted version.

## Conflict of Interest

The authors declare that the research was conducted in the absence of any commercial or financial relationships that could be construed as a potential conflict of interest.

## Publisher’s Note

All claims expressed in this article are solely those of the authors and do not necessarily represent those of their affiliated organizations, or those of the publisher, the editors and the reviewers. Any product that may be evaluated in this article, or claim that may be made by its manufacturer, is not guaranteed or endorsed by the publisher.
